# An Introduction to the OutSMART Cancer Serious Game: Current and Future Directions

**DOI:** 10.2196/56168

**Published:** 2024-05-29

**Authors:** Olufunmilola Abraham, Tyler J McCarthy

**Affiliations:** 1 Social and Administrative Sciences Division School of Pharmacy University of Wisconsin-Madison Madison, WI United States

**Keywords:** serious game, cancer, health education, adolescents, health behavior, United States, young people, adolescent, teenager, teenagers, cancer prevention, education, cancer risk, tool, OutSMART Cancer, innovative, game development, cancer awareness, prevention, wellness

## Abstract

Given that cancer is a challenging disease that plagues millions of individuals of all age groups and socioeconomic statuses globally, developmentally appropriate education is often lacking for young people, particularly adolescents. Increasing cancer awareness and prevention education among adolescents using innovative strategies, such as game-based learning, is critical in reducing the burden of this disease. Adolescents are understudied in the field of cancer prevention and control, yet vulnerable as they tackle creating life-long health behavior patterns. Targeting cancer prevention education for adolescents has the potential to support long-term healthy behavior and reduce their risk of cancer. This paper provides an overview of the Collaborative Research on MEdication use and family health (CRoME) Lab’s novel game-based cancer prevention education tool. OutSMART Cancer is an innovative, novel educational intervention in the form of a serious game. Serious games are educational tools that seek to impart knowledge and improve behaviors in their players. This game covers information related to breast cancer, colon cancer, and lung cancer. This viewpoint is a summary of the developmental process for the OutSMART Cancer game. We describe in detail the work preceding initial game development, the current version of the game, future directions for the game, and its educational potential. The long-term goal of OutSMART Cancer is to improve cancer awareness and knowledge regarding prevention behaviors in adolescents and support a lifetime of health and wellness.

## Introduction

The ubiquity of cancer in the United States denotes both the immense burden of the disease and the countless individuals devoted to spreading awareness. Although preventative and treatment-based measures have improved outcomes and reduced cancer deaths, the incidence of some of the most common cancers is on the rise [1]. Some studies have suggested that people in the United States lack the necessary knowledge and awareness of cancer, particularly those of lower socioeconomic status [2,3]. Similar results have been indicated in adolescents in the United Kingdom and the United States [4-7]. Thus, despite the prevalence of cancer awareness, there is still a salient and critical need to encourage cancer awareness and knowledge from a young age so that individuals can better understand the basic biological etiology of this disease and support life-long prevention behaviors.

For educational interventions to be successful, especially those involving complex and emotionally charged chronic conditions, they must be tailored to the intended audience. The Collaborative Research on MEdication use and family health (CRoME) Lab has a history of engaging with and educating adolescents and parents on health topics, such as cancer prevention, medication safety, and vaping prevention [8]. The CRoME Lab has co-designed serious games for adolescents and parents around prescription opioid medication safety [9]. Serious games are games designed with the characteristic purpose of imparting knowledge to the player, rather than merely providing a recreational experience [10]. In this viewpoint, we detail the work leading to the development of a cancer education serious game named OutSMART Cancer, the current state of the game, and future directions.

## Early Work With Youth Stakeholders

In 2020, our team began research in Wisconsin with middle and high schools, holding focus groups with 327 students and conducting a further survey with 235 students [11-13]. In one study, the CRoME Lab held 25 focus groups with 188 middle and high school students between the ages of 12 and 18 years [11]. Through exploring adolescent perceptions, we found that many adolescents were interested in learning about cancer, specifically, cancer prevention. Middle and high school students in this study recounted familiarity with basic cancer biology but indicated unfamiliarity with how to assess cancer risk and what behaviors they can institute to prevent cancer.

One survey study examined adolescent’s knowledge and attitudes toward cancer as well as the acceptability of a game-based learning approach for cancer education in homes, health care settings, and schools [12]. The survey responses reiterated the findings from the initial previous focus groups. Although most students expressed basic cancer knowledge, only 66% knew that individuals have some level of control over their cancer risk. Moreover, only 37.3% reported knowing how to lower their cancer risk, while 50% suggested they try to make healthy choices to reduce their risk. Study findings provided further evidence for the need to educate youth on cancer and its prevention. Most adolescents (82%) reported that they would accept the use of a game to help them learn about cancer.

These initial studies with adolescents informed the CRoME Lab’s design of the OutSMART Cancer gameplay book, which was further assessed through focus groups with adolescents. This gameplay book showed adolescents the initial conceptualization of OutSMART Cancer informed by the Cancer Clear and Simple Curriculum [14]. A total of 18 focus groups, comprising 139 adolescents, provided in-depth feedback on the playbook [13]. Adolescents indicated that they preferred a serious game over educational modalities, such as websites and videos. Our cumulative research to date has shown that a serious game that focuses on cancer knowledge and prevention and is tailored to the preferences of adolescents could be integral in improving adolescent cancer prevention behavior.

In 2023, OutSMART began early evaluation by adolescents and parents. This demonstration is currently unavailable to the public, as it is evaluated among key stakeholders—adolescents and parents. Informed consent has been collected from participants in each study related to the development and testing of this game. Findings from this study will result in an adapted version of the game, which will be used to evaluate efficacy and implementation.

## The OutSMART Cancer Game

**Figure 1:**
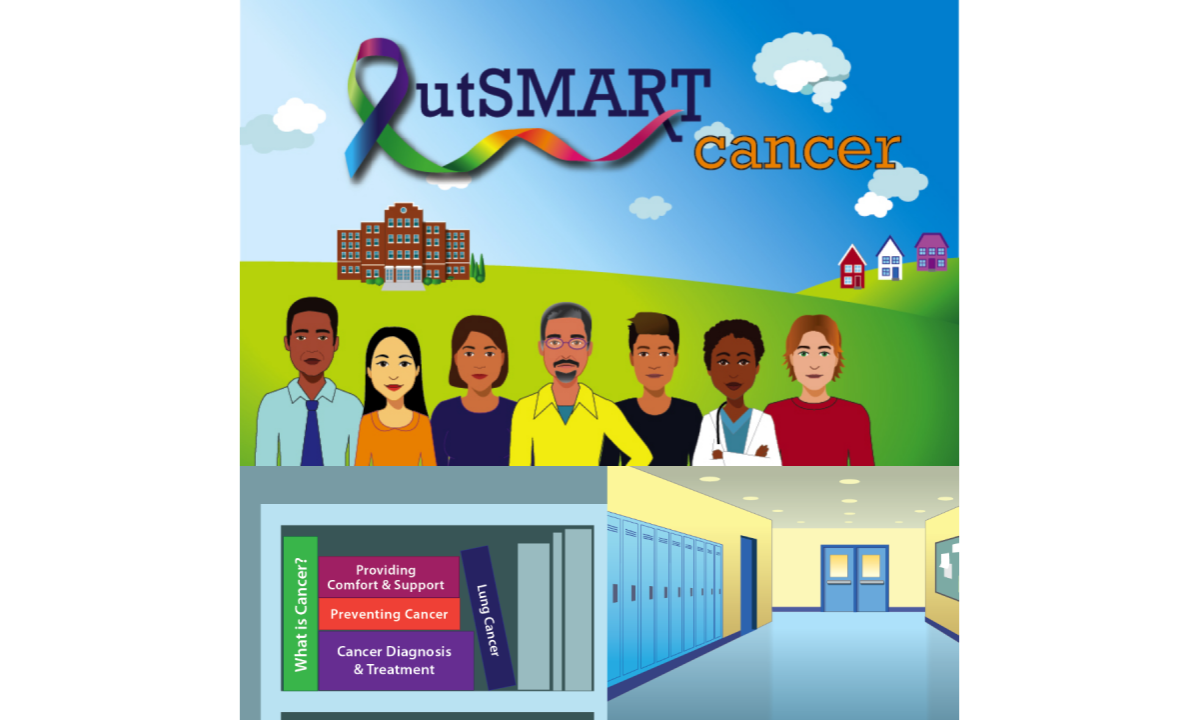
OutSMART Cancer gameplay.

**Figure 2:**
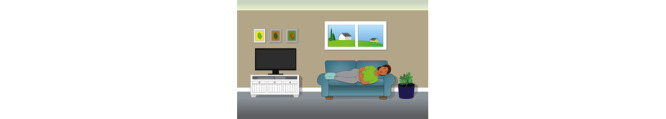
Screenshot of mom lying ill on the couch.

**Figure 3:**
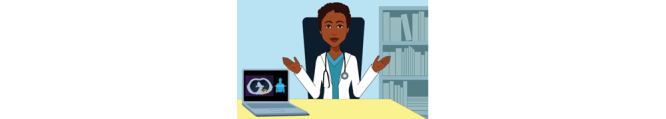
Screenshot of the doctor’s office scene.

**Figure 4:**
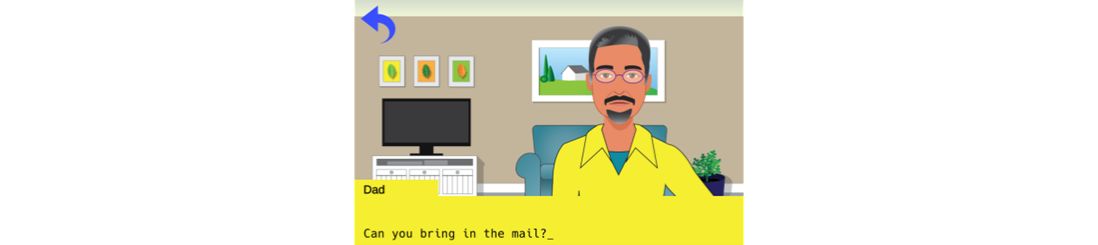
Colon cancer scene with father.

**Figure 5:**
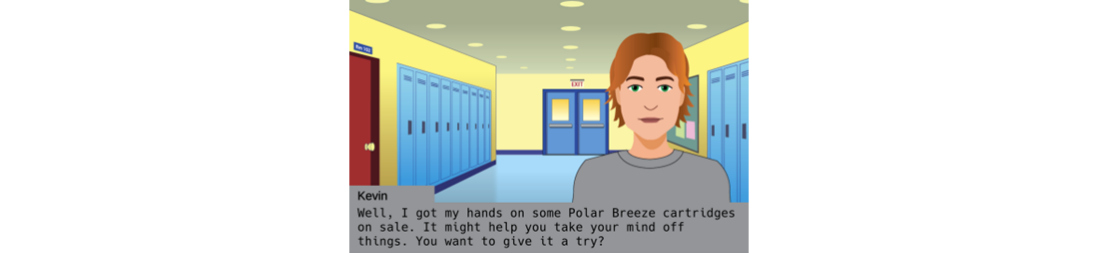
Peer offering vape to the player.

OutSMART is a web-based, computer videogame that presents 3 familiar, cancer-related scenarios in a narrative, choice-based format (Figures 1-5) [15]. It is built upon the Unity WebGL game engine and is currently optimized for browser gameplay on laptops and computers [16]. Within this game, players interact with the environment through a first-person perspective, taking the role of an adolescent. Players are faced with 3 scenarios that cover information related to breast, colon, and lung cancers. In each scene, players progress by clicking on pop-up bubbles, giving the player choices that move the storyline along. Players progress from one scene to the next once they have completed that scene’s storyline. After completion of a scene, players are taken to a map to choose the next available scene.

In the first scene, the player heads downstairs for a day at school to see their mother on the couch. After asking why she had not left for work, the mother tells the player that she is experiencing a painful lump in her breast alongside fatigue and will be going to her doctor that day. As the player offers to attend the appointment with her, the scene switches to the car ride to the doctor’s office. During this ride, the mother shares her family history of breast cancer and her anxiety. Later, in the doctor’s office, the player and their mother learn that the lump is cancerous. This level espouses key information, such as early warning signs of breast cancer, screening tools, basic cancer biology, and cancer stages.

In the second scene, the player brings mail inside for their father and discovers a letter from his doctor’s clinic encouraging him to schedule a colonoscopy. During this scene, the player tells their father about the letter and encourages him to schedule an appointment. However, at first, their father is apprehensive and talks of anxiety after his previous colonoscopy, which had uncovered polyps. After some conversation with their father, his attitude changes, and he decides to schedule a visit. This scene introduces colon cancer and screening strategies; it also introduces the player to the types of emotions that can act as barriers to screening.

In the final scene, the player goes to school and learns about cancer in a simulated classroom environment. Following class, their friend introduces them to a new person, who, when left alone with the player offers them a vape (electronic cigarette). Although peer pressure is evident, the player must refuse, articulating their own reasoning why they are choosing to protect their own health. This scene introduces players to the power of personal choice and how everyday choices can influence cancer risk.

## The Future of the OutSMART Cancer Game

Although preliminary evaluation and targeted adaptation of this game are ongoing, a simultaneous endeavor has begun to create a more adapted cancer-education game. This adaptation of OutSMART Cancer will emphasize the need for cancer awareness and targeted education among Black Americans and African immigrant youth and parents (OutSMART Cancer: Africana). The creation of OutSMART Cancer: Africana is responsive to the need for culturally competent cancer education for youth and African immigrant families living in the United States using culturally familiar language and imagery. Black Americans and African immigrants experience cancer and health care uniquely, requiring a tailored educational approach [17].

The initial intention is to widely disseminate this serious game through clinical settings, such as community pharmacies, primary care offices, and cancer clinics, as well as community settings, such as schools and community health organizations. Researchers aim for this intervention to be taken up by adolescents and their families across the United States. We expect that this game will eventually be publicly available on the internet for play on computers and mobile devices. The long-term goal of the OutSMART Cancer games is to facilitate family communication about cancer prevention, associated healthy behaviors, early detection, and cancer screening.

## Abbreviations

CRoME: Collaborative Research on MEdication use and family health
